# Complete genome sequences of the lytic bacteriophage PBM-3 and the avian pathogenic *Escherichia coli* strain PE144

**DOI:** 10.1128/mra.00873-25

**Published:** 2025-10-31

**Authors:** Jérémy D.R. Cherbuin, Jing Zhang, Muhammad Khan, Hira Niaz, Fazal Adnan

**Affiliations:** 1 Institute of Veterinary Bacteriology, Vetsuisse Faculty, University of Bern 27210, Bern, Bern, Switzerland; 2 Graduate School for Cellular and Biomedical Sciences, University of Bern 27210, Bern, Switzerland; 3 Multidisciplinary Center for Infectious Diseases (MCID), University of Bern 27210, Bern, Switzerland; 4 Atta ur Rahman School of Applied Biosciences (ASAB), National University of Sciences and Technology (NUST) 66959, Islamabad, Pakistan; Loyola University Chicago, Chicago, Illinois, USA

**Keywords:** bacteriophages, PacBio sequencing, avian pathogenic *Escherichia coli*, Pakistan

## Abstract

Avian pathogenic *Escherichia coli*, including strains that are resistant to antimicrobials, negatively impacts the poultry industry worldwide and the provision of animal protein to the people. Here, we report the complete genomes of the lytic bacteriophage PBM-3 and its bacterial host PE144, both isolated in Pakistan.

## ANNOUNCEMENT

Avian pathogenic *Escherichia coli* (APEC) causes colibacilloses (1) associated with major economic losses (2). Phages may assist in the control of infections with resistant APEC strains (3–5). We describe the genome sequences of the lytic bacteriophage PBM-3 and its APEC host PE144.

PE144 (O1:H7) was isolated from the liver of a 3-week-old chicken that succumbed to colibacillosis on a commercial farm in Rawalpindi, Pakistan, as described elsewhere (6). Genomic DNA was extracted from cells grown in lysogeny broth LB media at 37°C and 220 rpm overnight using the Wizard Genomic DNA Purification Kit (Promega).

Phage PBM-3 was isolated from 20 g of chicken bedding material, which was mixed with 40 mL of ¼ Ringer’s buffer, incubated overnight, and centrifuged at 5,000 g for 10 min. The supernatant was collected, passed through a 0.22 µm filter, plated on strain PE144 using LB double agar overlay, and incubated at 37°C overnight (7). Picked plaques were plaque-purified three times and enriched using the double-agar overlay method (7). Genomic DNA was extracted from high-titer stocks (>10^9^ pfu/mL) using the Monarch HMW DNA Extraction Kit (New England Biolabs).

DNA quantity and quality were evaluated using Qubit fluorometry (Thermo Fisher Scientific) and the Fragment Analyzer system (advanced analytical technologies), respectively. DNA was sheared by tagmentation, and fragments smaller than 3 kb were removed using 35% diluted AMPure beads, following the LongPlex Long Fragment Multiplexing Kit (seqWell). Library preparation was done with the LongPlex Kit and the SMRTbell Prep Kit 3.0 (Pacific Biosciences), followed by fragment analysis using Fragment Analyzer. Sequencing was conducted on the PacBio Revio system using one SMRT Cell 25M (30-hour movie time, Revio chemistry).

Default parameters were used for all software tools unless specified. Adapter trimming and sample demultiplexing were done using SMRT Link (v13.01), with adapter removal performed using the LongPlex Demultiplex Nextflow pipeline (seqWell). Raw reads were evaluated using Nanoplot (version 1.44.1) (8). The PE144 genome and the phage PBM-3 genome were assembled using Flye (version 2.9.4-b1799) (9, 10) and using QIAGEN CLC Genomic Workbench (version 25.0.1) (https://digitalinsights.qiagen.com), respectively. Quality of PE144 assembly was assessed by Quast (version 5.2.0) (11) and CheckM (12). Afterward, the PE144 genome was rotated to the first nucleotide of the start codon of the *dnaA* gene using Circlator (version 1.5.5) (13) and annotated using the NCBI Prokaryotic Genome Annotation Pipeline (PGAP version 6.10) (14). Sequence types and serotypes were identified using PubMLST (15) and serotype using SerotypeFinder (v2.0)  (16), respectively. The plasmid was assessed using PlasmidFinder (v2.1) (17, 18) (Table 1). The phage genome was annotated employing Pharokka (version 1.7.5) (19) and verified manually using Artemis (version 18.2.0) (20) and BLASTp (21).

**TABLE 1:** Sequencing statistics and genomic characteristics of APEC strains PE144 and bacteriophage PBM-3^*a*^^,^^*b*^

	APEC PE144		PBM-3
Location	Pakistan		Pakistan
Isolation source	Chicken liver		–
Total # of reads	269,240		7,076
Read N_50_ (bp)	7,528		9,215
ENA accession	ERR14801292		ERR15076224
GC content (%)	50.5		43.08
Replicon	Chromosome	pPE144_1	–
Assembly accession #	OZ287278.1	OZ287279.1	PV053165
Completeness	99.17%		
Total size (bp)	4,944,904	125,603	72,933
Coverage (×)	374	160	793
# CDSs	4,716	131	83
# tRNA genes	88	0	3
Circularity	Yes	Yes	No
MLST (Achtman)	95	–	–
Serotype	O1:H7	–	–
Plasmid	–	IncFIB	–

^*a*^ CDSs, coding DNA sequences; tRNAs, transfer RNAs; MLST, multilocus sequence typing.

^*b*^ “–” indicates not applicable.

**Fig 1:**
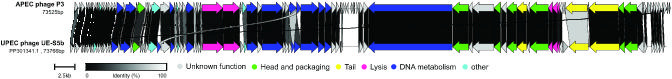
Genome alignment of phage PBM-3 and closest related phage UE-S5b (PP301341.1). Genome organization of the phage PBM-3 and UE-S5b visualized using clinker (23). PBM-3 had 98.89% genome sequence identity and 95% coverage with UE-S5b using BlastN (18).

The complete genome of PE144 had a 4,944,904 bp chromosome (374× coverage) harboring 4,716 coding DNA sequences (CDSs) and an IncFIB plasmid (160× coverage) of 125,603 bp harboring 131 CDSs (98.39% identity to GenBank AP001918). The PE144 genome had a GC content of 50.5%. The phage PBM-3 had a genome of 72,933 bp (793× coverage), a GC content of 43.08%, harbored 83 CDSs and three tRNA genes, and had 402 bp direct terminal repeats identified by Phageterm (22) (Fig. 1).

## Data Availability

The complete genome sequence of APEC strain PE144 has been deposited in the European Nucleotide Archive (ENA). PacBio reads are available under the Biosample SAMEA117970374 within the Bioproject PRJEB87885. The corresponding genome assembly is accessible under the accession number GCA_965645325.1 (OZ287278.1 for the chromosome and OZ287279.1 for the plasmid) and the raw PacBio HiFi sequencing reads are under accession number ERR14801292. The SRA study number is ERP171074 and the experiment number is ERX14207129. The annotated assembly of phage PBM-3 can be accessed under the accession number PV053165 and the raw PacBio HiFi sequencing reads under the accession number ERR15076224 (experiment number ERX14480869) in the Bioproject PRJEB87885 and Biosample SAMEA118363535. The SRA study number is ERP171074.
